# Predictors of Uncertainty and Unwillingness to Receive the COVID-19 Vaccine in Men Who Have Sex with Men in France

**DOI:** 10.3390/ijerph19095372

**Published:** 2022-04-28

**Authors:** Youssoufa M. Ousseine, Sophie Vaux, Stéphanie Vandentorren, Isabelle Bonmarin, Karen Champenois, Nathalie Lydié, Annie Velter

**Affiliations:** 1 Santé Publique France, French National Public Health Agency, 94415 Saint-Maurice, France; sophie.vaux@santepubliquefrance.fr (S.V.); stephanie.vandentorren@santepubliquefrance.fr (S.V.); isabelle.bonmarin@santepubliquefrance.fr (I.B.); nathalie.lydie@santepubliquefrance.fr (N.L.); annie.velter@santepubliquefrance.fr (A.V.); 2 Université de Bordeaux, INSERM UMR 1219, PHAreS Team, 33000 Bordeaux, France; 3 Université de Paris, INSERM, IAME, 75006 Paris, France; karen.champenois@gmail.com; 4 Aix Marseille Univ., INSERM, IRD, SESSTIM, Sciences Economiques & Sociales de la Santé & Traitement de L’Information Médicale, ISSPAM, 13005 Marseille, France

**Keywords:** vaccine-hesitancy, uncertainty, unwillingness, COVID-19 vaccine, men who have sex with men, health literacy, social inequalities

## Abstract

The development of vaccines against COVID-19 has given hope to populations. Public acceptability of vaccination is a major driver in containing the disease. However, in marginalized and stigmatized populations, uncertainty and unwillingness may be a challenge. This study aimed to analyze the factors associated with uncertainty and unwillingness to vaccinate against COVID-19 in men who have sex with men (MSM) living in France. The data used came from Rapport au Sexe (ERAS) 2021, a voluntary, cross-sectional, anonymous, self-administered, online survey conducted from 26 February to 11 April 2021. Among the 15,426 respondents included in the analysis, 60.5% were willing to vaccinate (these included persons already vaccinated), 17.5% were not, and 22% were uncertain. Factors independently associated with uncertainty and unwillingness were lower education level, low health literacy level, financial hardship, being under 30 years of age, and living in a rural area. HIV-positive MSM were less likely to report vaccination uncertainty and unwillingness than HIV-negative MSM and those with unknown serostatus. Although more impacted by COVID-19, socioeconomically vulnerable MSM were the sub-group most unwilling to vaccinate. To improve acceptability of COVID-19 vaccination in MSM, policy makers and researchers must increase access to and understanding of medical information by considering the general public’s health literacy when developing information sources. Moreover, a dedicated global care approach, which ensures these populations can be reached, is necessary.

## 1. Introduction

In December 2019, an unusually high number of cases of viral pneumonia were reported in Wuhan, China. The rapid spread of the associated virus SARS-CoV-2, and the consequent infection COVID-19, quickly became a concern for global health authorities. The WHO declared a pandemic on 11 March 2020. The lack of a cure or vaccine at the time, and the high transmissibility of the infection led to protective measures, such as national lockdowns and curfews. Meanwhile, scientists put all their efforts into developing a COVID-19 vaccine. For the first time in modern medical history, mRNA-based vaccines were rapidly developed in less than a year. By the end of 2020, studies had shown the safety and efficacy of the Moderna and Pfizer/BioNtech mRNA vaccines [1,2]. Furthermore, the AstraZeneca vaccine (ChAdOx1 nCoV-19 vaccine-AZD1222) using viral vector technology had shown positive safety and efficacy results [3]. A study confirmed the effectiveness of these COVID-19 vaccines [4].

In France, the vaccination strategy began on 27 December 2020; it consisted of phased vaccination, first prioritizing the elderly and/or those weakened by morbidity factors, in order to protect people most likely to develop a serious form of the disease. Priority was also given to health professionals. Only from 12 May 2021 was vaccination extended to people under 50 years old.

Vaccination is a crucial driver in containing the COVID-19 pandemic. However, unwillingness to be vaccinated is widespread worldwide [5]. In June 2020, intention to vaccinate against COVID-19 differed greatly between countries. France, Poland, and Russia had the lowest rates worldwide (58.9%, 56.3%, and 54.9%, respectively) [6]. Previous studies showed gender differences in unwillingness to be vaccinated against COVID-19, with significant unwillingness in women [7,8]. Several factors have been associated with uncertainty or unwillingness, including age, education level, socioeconomic status, perceived seriousness of COVID-19, and sexual orientation [6,7,9,10,11]. For example, in Puerto Rico, gay self-identity was associated with greater intention to be vaccinated [9].

Marginalization and systematic discrimination of sexual minorities led to inequalities of all kinds. The COVID-19 health crisis has had a greater impact on sexual minorities than the general population, particularly men who have sex with men (MSM) [12]. It has highlighted pre-existing vulnerabilities specific to MSM in terms of health, isolation, socioeconomics, and mental health [12,13,14,15,16]. These vulnerabilities may have exacerbated stigma and internalized homonegativity (feelings of guilt, inferiority, and low self-esteem), two factors associated with low utilization of care and poor preventive behaviors [17,18].

In addition, the rapid development of COVID-19 vaccines, and the divergent medical information regarding these vaccines and the pandemic, may have triggered or reinforced COVID-19 vaccine hesitancy in people with low health literacy [19,20]. While several studies have documented factors associated with intention to vaccinate in the general population, few have focused on sexual minorities [21,22]. Knowing that the binary consideration of gender and the omission of sexual orientation in access to care could be a source of inequality, we must make efforts to build an inclusive environment for equity. This study aimed to investigate factors associated with intention to vaccinate against COVID-19 in the MSM population in France.

## 2. Materials and Methods

### 2.1. Study Design and Participants

We used data from Rapport au Sexe 2021, a large, cross-sectional online survey of MSM in France conducted between 26 February and 11 April 2021. Respondents who self-identified as homosexual or bisexual or have had sex with a man in their lifetime are considered MSM. The survey was anonymous, self-administered, and voluntary. Participants were recruited through different digital media. Banners were posted directly on gay dating websites, gay geolocation dating applications, gay affinity news sites, and social media networks (Facebook). They were also posted via programmatic platforms targeting men aged 18 years old and over and on browsing pages containing keywords related to homosexuality and male dating. By clicking on these banners, people were directed to the survey site, where information about its objectives was presented as well as the conditions of participation and data confidentiality. By clicking on a button containing the text “I have read and understood the information above”, the participant provided informed consent and was directed to the online questionnaire. No IP address was collected. No financial incentive was given. The only inclusion criterion was being aged 18 years and older.

### 2.2. Data Collection

#### 2.2.1. Outcome: Intention to Vaccinate against COVID-19

Intention to vaccinate against COVID-19 was measured using the following question: “Do you intend to be vaccinated against Coronavirus? Yes/I have already been vaccinated against the Coronavirus in the last few weeks/I do not know/No”. Respondents were then classified into three groups: willing (already vaccinated or intended to), uncertain (were unsure about vaccination), or unwilling (did not want to be vaccinated).

#### 2.2.2. Independent Variables

The following socioeconomic and demographic characteristics were collected: age, place of birth, area of residence, having a steady relationship with a man, education level, occupational situation, perceived financial situation, and health literacy. Perceived financial situation was measured with the question *“Would you say that financially…” (‘you are comfortable’; ‘you get by’; ‘you have to be careful’; ‘you find it difficult to make ends meet’; and ‘you can’t make ends meet without incurring debt’*). Response categories were merged to form a three-level variable: comfortable (which covered ‘comfortable’ and ‘you get by’; need to be careful; difficulty and debt).

Health literacy was evaluated using the Health Literacy Questionnaire (HLQ) scale ‘having sufficient information to manage my health’ [23,24]. This scale contains four items, each scored on a 4-point Likert scale. The total score is calculated as the average of the four item scores, and ranges from 1 to 4. Participants were categorized into two groups: low (score ≤ 2.8 1st quartile) and adequate (score > 2.8) health literacy.

Respondents also reported their vaccination history (hepatitis A and hepatitis B) and their HIV status. Furthermore, COVID-19-related information was collected, such as COVID-19-like symptoms or signs (yes/no) and diagnosed with COVID-19 (yes/no). In addition to these individual factors, respondents could report the reasons for their unwillingness to be vaccinated from a list of suggestions (COVID-19 vaccines are unsafe and side effects are not really known, doubts about vaccine effectiveness, COVID-19 is not a very dangerous disease, vaccine hesitant in general, not liking injections, other reasons).

### 2.3. Statistical Analysis

We performed univariate analyses to describe respondents’ characteristics. Median and interquartile range (IQR) were computed for continuous variables. Categorical variables were expressed as proportions. Chi-squared tests were used to compare categorical data.

To identify factors associated with intention to vaccinate against COVID-19, multinomial logistic models were used. A backward procedure was employed to select statistically significant factors in the multivariate models (entry threshold, *p* < 0.20). Only factors with a *p* < 0.05 were kept in the final multivariate model. Statistical analyses were performed using Stata software version 15 (StataCorp, College Station, TX, USA).

## 3. Results

**Figure 1:**
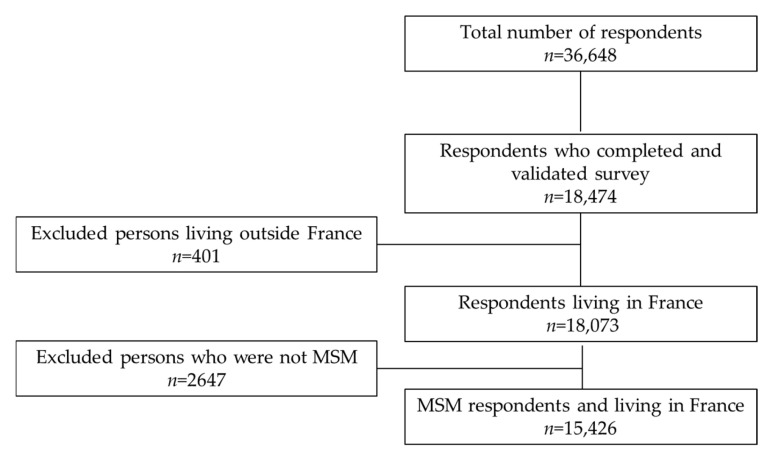
Participant flow chart.

Of the 36,648 people who started the questionnaire, 18,474 (50%) completed and validated it (Figure 1). Respondents who discontinued the questionnaire were younger on average than those who completed it (32.2 vs. 34.7 years). They had lower than the upper secondary school certificate (39.8 vs. 32.1) and were more heterosexual or refused to self-identify (54.8 vs. 21.3) than respondents completed questionnaire. Among the latter, 3048 were excluded because they lived outside France (401) or were not MSM (2647). The majority of the sample (80%) had connected to the survey via social networks.

Most of the respondents were born in France (94.6%), lived in an urban area (82.8%), and had a third-level education level (68.2%). Just under a third (31.1%) had a low level of health literacy, and 14% perceived their financial situation as difficult. With regard to COVID-19, 42.5% of respondents reported having COVID-19-like symptoms or signs, and 9.7% had been diagnosed with the disease. At the time of the study (February to April 2021), 60.5% of respondents were already COVID-19 vaccinated or intended to vaccinate, 17.5% did not intend to vaccinate, and 22% were uncertain about vaccinating (Table 1).

### Factors Associated with Uncertainty and Unwillingness to Receive the COVID-19 Vaccine

In univariate analyses, uncertainty and unwillingness were associated with age, occupation, area of residence (i.e., urban versus rural) and vaccination for hepatitis A and B diseases (Table 2).

MSM aged 25–29 years old were more likely to report unwillingness to vaccinate (22.3%) than other age groups (18–24 years: 20.7%, 30–44 years: 18%, and ≥45 years: 10.2%, *p* < 0.001). Unemployed, inactive, and retired MSM were also more likely to be unwilling to vaccinate. In addition, the relationships between education level, health literacy, financial situation, and intention to vaccinate revealed a socioeconomic gradient. More specifically, MSM with a lower level of education (i.e., <third-level), a lower level of health literacy, and those perceiving their financial situation as difficult, were all more likely to be unwilling to vaccinate or uncertain about it. COVID-19 diagnosis and having had COVID-19-like symptoms were both negatively associated with uncertainty and unwillingness to vaccinate. Finally, HIV-negative MSM using PrEP and HIV-positive MSM were less likely to report uncertainty and unwillingness to vaccinate than HIV-negative MSM or those with unknown serostatus.

In the multivariate analysis, the factors independently associated with uncertainty and unwillingness to vaccinate were the same (Table 3). More specifically, MSM 29 years old and under (18–24 years: Ora = 1.34, 95%CI [1.15–1.56]; 25-29 years: Ora = 1.41, 95%CI [1.24–1.61]), those residing in rural areas (Ora = 1.71, 95%CI [1.52–1.91]), and persons born in France (Ora = 1.67, 95%CI [1.32–2.12]) were more likely to be unwilling to vaccinate against COVID-19. Furthermore, participants with less than third-level education (Ora = 2.11, 95%CI [1.87–2.38] and Ora = 3.09, 95%CI [2.72–3.51], for upper secondary school certificate and lower, respectively), those with low health literacy (Ora = 1.19, 95%CI [1.08–1.32]), and persons who just got by financially (Ora = 1.65, 95%CI [1.49–1.84]) or had a difficult financial situation (Ora = 2.31, 95%CI [2.02–2.63]) were all more likely to be unwilling to vaccinate against COVID-19. The effect sizes of less than third-level education (Ora = 2.12, 95%CI [1.88–2.39] vs. Ora = 3.09, 95%CI [2.72–3.51]) and having a difficult financial situation (Ora = 1.61, 95%CI [1.42–1.83] vs Ora = 2.31, 95%CI [2.02–2.63]) were lower for persons who were uncertain than those who were unwilling. MSM HIV negative using PrEP and HIV positive MSM were less uncertain and less unwilling to vaccinate against COVID-19.

**Figure 2:**
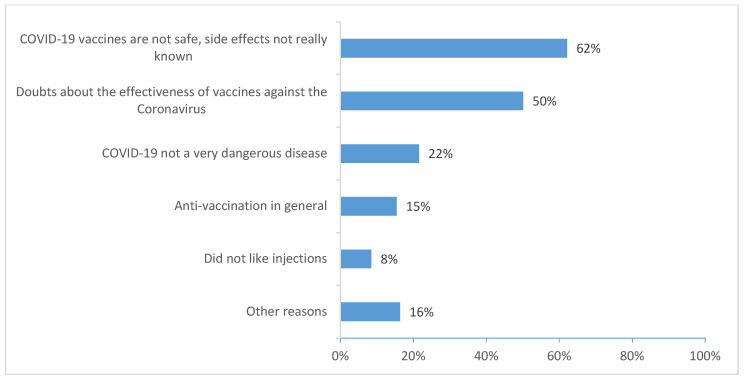
Reasons for unwillingness to vaccinate against COVID-19 (ERAS 2021 *n* = 2692).

The reasons MSM reported for their unwillingness to vaccinate against COVID-19 were related to the safety and unknown adverse effects of vaccines (62%), doubts about their effectiveness (51%), perceiving COVID-19 not to be dangerous (24%), and being against vaccination in general (16%) (Figure 2).

## 4. Discussion

People who are more socially disadvantaged are at a disproportionately greater risk of COVID-19 as well as developing severe forms and dying [25]. The current COVID-19 period has seen sexual minorities, particularly MSM, face a deterioration of their socioeconomic status [12]. The rapid availability of vaccines against COVID-19 has given hope to different populations everywhere. This availability has however been accompanied by growing public vaccine refusal and hesitancy.

In our study, 60% of MSM had already vaccinated against COVID-19 or intended to do so. This rate is very close to those found for the French general population by Lazarus et al. (58.9%) in June 2020, and Neumann-Böhme et al. (62%) in April 2020 [6,26]. A study conducted between April and May 2020 in 5018 participants from the French general population reported that 76% intended to vaccinate (39.9% “probably” and 36.1% “certainly”) [7]. However, in April 2021, the French national survey Coviprev reported a slightly high COVID-19 vaccination uptake rate (68%) [27]. These marginal differences in intention to vaccinate might be related to the different timing of the surveys and also to differences in survey methodology. The ERAS 2021 survey was conducted after vaccinations had begun. They may also be related to the fact that adverse effects after vaccination created a great deal of controversy with the subsequent reluctance by some to vaccinate. A previous study showed that intention to vaccinate against COVID-19 varied according to survey date [7].

Our study showed that the factors associated with uncertainty were the same as those associated with unwillingness. Age and vaccination history were negatively associated with COVID-19 vaccine uncertainty and unwillingness. Older MSM and those previously vaccinated for hepatitis A and/or hepatitis B were less hesitant about vaccination. These results are consistent with findings in previous studies [6,7,28]. One possible explanation is that the possibility to vaccinate was extended to people in France under 50 years of age after this survey was implemented. Unlike Alleaume et al., in our study, living with a partner was associated with a greater likelihood of COVID-19 vaccine uncertainty and unwillingness. Our result regarding the role of social density—measured by several indicators, including area of residence (metropolitan versus urban)—on the likelihood of vaccination, reflects previous research studying the impact on vaccination against COVID-19 or influenza [29]. In addition, this could also be related to reasons of social gradient and isolation. People living in rural areas may be less concerned about the issue and may perceive the disease to be less severe than people in urban areas.

HIV-negative MSM using PrEP and HIV-positive MSM were less likely to be uncertain about vaccination or unwilling to vaccinate. Between February and April 2021, HIV-positive individuals were one of the high-risk populations given vaccination priority in France. Governmental recommendations may therefore have played a role in intention to vaccinate in HIV-positive MSM. For MSM using PrEP, their positive attitude about COVID-19 vaccination might be related to their risk behaviors and lifestyle. The reasons to vaccinate are to protect oneself and to have a positive impact on others within one’s community [29]. In addition, having had COVID-19-like symptoms was negatively associated with vaccine refusal. Being confronted with the reality of the disease may influence the acceptance of the COVID-19 vaccine.

Our results stress the existence of a socioeconomic gradient regarding the intention to vaccinate against COVID-19. Less that third-level education, perceived financial hardship, and low health literacy were associated with a greater likelihood of vaccine hesitancy and unwillingness. With respect to education level and health literacy, our results are consistent with recent studies on intention to vaccinate against COVID-19, as reported in a systematic review [28,30,31]. Difficulties in understanding scientific information—especially complex COVID-19-related information—is a barrier to informed decision-making. Our results reflected those from other studies showing that the two most-cited reasons for unwillingness to vaccinate against the disease were that “COVID-19 vaccines are not safe, side effects not really known (62%)” and “doubts about the effectiveness of COVID-19 vaccines” (50%) [5]. These reasons reflect a poor understanding of health information because current COVID-19 vaccines are considered safe and effective despite moderate side effects [1,2,3]. In terms of financial position, people with financial difficulties often do not have sufficient access to prevention and care services. Other studies have shown that low income is associated with unwillingness to vaccinate against COVID-19 [5,7].

Policy makers and institutions implementing immunization programs face major challenges, including making scientific information accessible, clear, and easy to understand for better adherence by the most vulnerable populations. In addition, providing outreach for population who are the most hesitant about vaccination should be considered, especially for disadvantaged populations. The scientific literature has shown that several actions are favorable to encourage disadvantaged people to be vaccinated. This is the case, for example, of public vaccination of high-profile individuals and community leaders [32]. In addition, mobile vaccination unit programs make it possible to go directly to the vulnerable populations in order to combat misinformation, to convince them and to get them vaccinated [33]. In France, a mobile unit for COVID-19 vaccination has given satisfactory results [34]. Other actions such as the translation of information documents into several languages and the writing plain language have been implemented within the national public health agency in France.

This study has limitations. A low response rate (50% of questionnaires completed and validated), often including heterosexuals not concerned by the study. The methodology of an online survey did not reach MSM who do not use the web. However, given the importance of the internet and social networks in MSM lifestyles, an online survey remains the most relevant and cost-effective methodology for reaching this hard-to-reach population [35]. Sexual, declarative, voluntary surveys—such as ERAS—tend to over-represent men with the strongest gay self-identity [36]. Therefore, our results cannot be generalized to the entire population of MSM living in France for lack of representativeness of the sample. Indeed, the absence of both a sample frame and controls during the inclusion process means that our results cannot be extrapolated to the entire MSM population [37]. Having said that, recruitment via social networks allowed us to diversify the sociodemographic and affinity profiles, and to include MSM who were more distant from the gay community, and persons more economically disadvantaged. Moreover, the timing of the survey (February to April 2021) may have led to an underestimation of overall vaccination acceptance because of misinformation and anti-vaccination campaigns which were prevalent on social networks. These elements may have made it more difficult for people to decide to vaccinate. Furthermore, the suspension of the AstraZeneca vaccine during the survey period may have increased vaccine-hesitancy. However, it should be noted that our vaccine willingness and hesitancy rates were close to those reported in the Coviprev survey [27]. The use of self-perceived measures such as HL could lead to bias. However, the HL had good reliability. Finally, the ERAS survey was not developed specifically to assess vaccine-hesitancy and therefore did not assess all factors that may influence uncertainty and unwillingness, such as perceived current health status, perceived susceptibility of contracting COVID-19, perceived severity of the disease, and perceived benefits of and perceived barriers to vaccination. However, the factors observed in this specific population are consistent with the literature.

## 5. Conclusions

Uncertainty and unwillingness to be vaccinated is a major problem for vaccination programs. For a global pandemic, such as the COVID-19 crisis, rapid herd immunity must be achieved through mass vaccination. In order to increase vaccination coverage, it is necessary to understand the factors associated with vaccine-hesitancy. More impacted by COVID-19, socioeconomically vulnerable persons in our study were the MSM most reluctant to vaccinate. Vaccine prevalence was lower in vulnerable populations than in the general French population (74.9% with at least one dose vs. 89.3% in the general population, and 72.7% with two doses vaccination vs. 87.3% in the general population) [38]. For a virus that continues to evolve, a 60% vaccination rate for MSM remains low.

Our results will allow decision makers to do targeted work to convince the hesitant and reach out to the most disadvantaged populations (socioeconomically vulnerable MSM and those with a low health literacy level) are often the furthest from the healthcare system. In addition, our results will help improve recall campaigns. To improve acceptability of COVID-19 vaccination in MSM, policy makers and researchers must improve access to and understanding of medical information, by considering health literacy when developing information sources. Moreover, a dedicated global care approach, which ensures these populations can be reached is necessary.

## Figures and Tables

**Table 1:** Participants’ characteristics (*n* = 15,426).

	*n*	%
Age, median [IQR] (years)	33 [25–44]	
18–24	3828	24.8
25–29	2557	16.6
30–44	5238	34
≥45	3803	24.7
Place of birth		
Outside France	834	5.4
France	14,592	94.6
Area of residence		
Rural	2653	17.2
Urban	12,773	82.8
Education level		
Lower than upper secondary school certificate	2328	15.1
Upper secondary school certificate	2583	16.7
Third-level	10,515	68.2
Health literacy		
Low	4804	31.1
Adequate	10,622	68.9
In a steady relationship with a man		
No (Single/separated/other)	8207	46.8
Yes (Married/cohabitation/civil partnership)	7219	53.2
Occupation		
Employee, self-employed	10,164	65.9
Unemployed, not active or retired	2324	15.1
Student	2938	19
Perceived financial situation		
Comfortable	8978	58.2
Need to be careful	4294	27.8
Difficulty and debt	2154	14
Biomedical status		
HIV negative and PrEP user	1504	9.7
HIV negative	10,002	64.8
HIV positive	1023	6.6
Unknown HIV status	2897	18.8
Had COVID-19-type symptoms		
No	8877	57.5
Yes	6549	42.5
Diagnosed with COVID-19		
No	13,937	90.3
Yes	1489	9.7
Vaccination history (vaccinated against Hepatitis A or B)		
No	5279	34.2
Yes	10,147	65.8
Intention to vaccinate against COVID-19		
Willing (already vaccinated and intended)	9335	60.5
Uncertain	3399	22
Unwilling	2692	17.5

**Table 2:** Univariate analyses of factors associated with intention to vaccinate against COVID-19 (*n* = 15,426).

	Intention to Get Vaccinations against COVID-19						
	Willing *n* = 9335		Uncertainly *n* = 3399		Unwillingness *n* = 2692		
	*n*	%	*n*	%	*n*	%	*p*-Value
Age (years)							***
18–24	2008	52.5	1029	26.9	791	20.7	
25–29	1391	54.4	595	23.3	571	22.3	
30–44	3077	58.7	1218	23.3	943	18	
≥45	2859	75.2	557	14.6	387	10.2	
Place of birth							***
Outside France	595	71.3	149	17.9	90	10.8	
France	8740	59.9	3250	22.3	2602	17.8	
Area of residence							***
Rural	1287	48.5	686	25.9	680	25.6	
Urban	8048	63	2713	21.2	2012	15.8	
Education level							***
Lower than upper secondary school certificate	1010	43.4	627	26.9	691	29.7	
Upper secondary school certificate	1228	47.5	716	27.7	639	24.7	
Third-level	7097	67.5	2056	19.6	1362	13	
Health literacy							***
Low	2679	55.8	1179	24.5	946	19.7	
Adequate	6656	62.7	2220	20.9	1746	16.4	
In a steady relationship with a man							***
No (Single/separated/other)	4724	57.6	1903	23.2	1580	19.3	
Yes (Married/cohabitation/civil partnership)	4611	63.9	1496	20.7	1112	15.4	
Occupation							***
Employee, self-employed	6316	62.1	2155	21.2	1693	16.7	
Unemployed, not active or retired	1304	56.1	506	21.8	514	22.1	
Student	1715	58.4	738	25.1	485	16.5	
Perceived financial situation							***
Comfortable	6021	67.1	1771	19.7	1186	13.2	
Need to be careful	2328	54.2	1065	24.8	901	21	
Difficulty and debt	986	45.8	563	26.1	605	28.1	
Biomedical status							***
HIV negative and PrEP user	1134	75.4	250	16.6	120	8	
HIV negative	5946	59.4	2253	22.5	1803	18	
HIV positive	801	78.3	134	13.1	88	8.6	
Unknown HIV status	1454	50.2	762	26.3	681	23.5	
Vaccination history (vaccinated against Hepatitis A or B)							***
No	2739	51.9	1353	25.6	1187	22.5	
Yes	6596	65	2046	20.2	1505	14.8	
Had symptoms or signs of COVID-19							***
No	5290	59.6	1944	21.9	1643	18.5	
Yes	4045	61.8	1455	22.2	1049	16	
Diagnosed with COVID-19							***
No	8351	59.9	3105	22.3	2481	17.8	
Yes	984	66.1	294	19.7	211	14.2	

*** *p*-value < 0.001.

**Table 3:** Multivariate analyses using multinomial logistic models of factors associated with intention to vaccinate against COVID-19 (*n* = 15,426).

	Uncertainly vs. Acceptance		Unwillingness vs. Acceptance	
	ORa	95%CI	ORa	95%CI
Age (years)				
18–24	1.26	1.09–1.45	1.34	1.15–1.56
25–29	1.10	0.98–1.24	1.41	1.24–1.61
30–44	1		1	
≥45	0.44	0.39–0.50	0.36	0.31–0.42
Place of birth				
Outside France	1		1	
France	1.33	1.10–1.61	1.67	1.32–2.12
Area of residence				
Rural	1.40	1.26–1.56	1.71	1.52–1.91
Urban	1		1	
Education level				
Lower than upper secondary school certificate	2.12	1.88–2.39	3.09	2.72–3.51
Upper secondary school certificate	1.78	1.60–1.99	2.11	1.87–2.38
Third-level	1		1	
Health literacy				
Low	1.21	1.11–1.32	1.19	1.08–1.32
Adequate	1		1	
In a steady relationship with a man				
No (Single/separated/other)	1		1	
Yes (Married/cohabitant/civil partnership)	0.86	0.79–0.94	0.79	0.72–0.87
Occupation				
Employee, self-employed	1		1	
Unemployed, not active or retired	0.98	0.86–1.11	1.08	0.95–1.23
Student	0.83	0.72–0.96	0.66	0.56–0.77
Perceived financial situation				
Comfortable	1		1	
Need to be careful	1.39	1.26–1.52	1.65	1.49–1.84
Difficulty and debt	1.61	1.42–1.83	2.31	2.02–2.63
HIV status				
HIV negative and PrEP user	0.74	0.62–0.89	0.45	0.36–0.57
HIV negative	0.98	0.88–1.10	0.93	0.83–1.05
HIV positive	0.57	0.46–0.71	0.44	0.34–0.58
Unknown HIV status	1		1	
Vaccination history (vaccinated against Hepatitis A or B or HPV)				
No	1		1	
Yes	0.74	0.67–0.80	0.67	0.61–0.74
Had symptoms or signs of COVID-19				
No	1		1	
Yes	0.93	0.85–1.01	0.81	0.74–0.89

## Data Availability

The datasets used and/or analyzed during the current study are available on request from the corresponding author.
